# Tubal patency during the menstrual cycle and during treatment with hormonal contraceptives: a pilot study in women

**DOI:** 10.1177/0284185116679457

**Published:** 2016-11-17

**Authors:** Jeffrey T Jensen, Eva Patil, Jacqueline Seguin, Amy Thurmond

**Affiliations:** 1 Oregon Health & Science University, Department of Obstetrics and Gynecology, Portland, OR, USA; 2 Womens Imaging & Intervention Center, Lake Oswego, OR, USA

**Keywords:** Hysterosalpingography, tubal sterilization, menstrual cycle, oral contraception, Fallopian tubes

## Abstract

**Background:**

Hysterosalpingram (HSG) evaluation of tubal patency is typically performed in the follicular phase, but data to support this timing are lacking.

**Purpose:**

To determine whether menstrual cycle phase or hormonal treatments affect observation of tubal patency during HSG.

**Material and Methods:**

Ten participants underwent repeated HSG examinations: during the follicular and luteal phase of a natural menstrual cycle; 30 days following continuous administration of a combined oral contraceptive (COC); and 30 days after an intramuscular injection of depo medroxyprogesterone (DMPA) acetate. Participants with tubal blockade following DMPA had a fifth HSG 30 days following a second course of COCs. The primary outcome was tubal patency.

**Results:**

All 10 participants demonstrated bilateral tubal patency (BTP) on at least one HSG examination during the study. One participant showed bilateral functional occlusion (FO) during the follicular phase examination, but BTP with the luteal phase, COC cycle, and DMPA exams. One participant with BTP discontinued participation and nine completed the COC HSG exam with BTP in seven, and one each with bilateral or unilateral FO. Seven participants completed the DMPA HSG with BTP in six and unilateral FO in one; BTP was seen in the final HSG after restarting the COC.

**Conclusion:**

This pilot study supports the luteal phase of natural cycles as the optimum time for evaluation of tubal patency. The occurrence of functional occlusion of the fallopian tube on HSG examination performed during the follicular phase and following contraceptive steroid treatment supports a role of hormonal action on the utero-tubal junction.

## Introduction

Hysterosalpingography, a minimally invasive radiologic technique to image the female reproductive tract, provides detailed information about the endometrial cavity and Fallopian tube, structures not well-characterized on routine pelvic ultrasound. Common indications for hysterosalpingography include evaluation of tubal patency during an infertility workup or confirmation of occlusion following a permanent contraception procedure.

Clinicians typically perform the hysterosalpingogram (HSG) exam during the early follicular phase. Early menstrual cycle timing has certain advantages; the woman cannot be pregnant and the endometrium is thin facilitating visualization of the cavity. However, when the objective is evaluation of tubal patency, no recent studies have evaluated whether menstrual cycle timing or hormonal therapy influences success. In 1931, Mathieu published a case series of women with hydrosalpinx diagnosed by HSG. Although the paper references that HSG had become well-established by 1927, no details are presented regarding timing of the procedure (1). Lindhal and Helander published the first report to describe HSG performed at various stages during the menstrual cycle in 1960 (2). In this report, HSG was performed on women of proven fertility who had recently undergone uncomplicated first trimester abortion. The authors performed 511 HSG exams at different stages of the menstrual cycle and observed unilateral or bilateral blockade in 49 cases (0% early proliferative; 22.6% late proliferative, 5.7% early secretory; 4.8% mid-secretory, 11.3% late secretory, and 11.6% menstrual). Although all of the early proliferative phase cases showed bilateral tubal patency (BTP), only 8% of the 511 exams were performed in the proliferative phase (early [n = 11] and late [n = 31]), and the authors recommended the early secretory phase as the best stage for tubal evaluation. Given the absence of other gynecologic pathology or history of infection in the women, these obstructions were considered functional rather than anatomic. However, these authors did not repeat the exams in women with tubal non-patency at a different time in the cycle and cycle timing was evaluated by basal temperature and endometrial biopsy.

Our interest in non-surgical permanent contraception for women led us to consider whether menstrual cycle timing could affect the delivery of agents administered transcervically into the Fallopian tubes. Quinacrine sterilization, typically performed in the follicular phase has a failure of about 27% with one application (3). Timing might also explain some cases of unilateral or bilateral failed microinsert placement during hysteroscopic permanent contraception (4).

Functional occlusion is defined as an obstruction of the Fallopian tube at the utero-tubal junction observed during HSG that is not explained by a true anatomic blockade or presence of an obstructing material (5). Functional tubal occlusion may be cycle-dependent or related to estrogen and progesterone-receptor mediated events that could be influenced by hormonal contraception (6). To address the hypothesis that cycle stage or contraceptive steroid hormones affect the probability of observing BTP on HSG, we performed a pilot study where participants underwent repeat HSG examinations at various points during the menstrual cycle, and during treatment with a combined oral contraceptive (COC) and depomedroxyprogesterone acetate (DMPA).

## Material and Methods

We conducted an open-label pilot study at Women’s Imaging and Intervention (WI&I) and Oregon Health & Science University (OHSU) in Oregon, USA. The Institutional Review Board at OHSU approved the study protocol. We performed all screening procedures at OHSU and HSG exams at WH&I.

We recruited healthy reproductive age women aged 18–40 years with normal menstrual cycles not using hormonal contraception or an IUD. Key exclusions included pregnancy, contraindications to use of combined hormonal contraception, undiagnosed genital bleeding, and sensitivity to radiologic contrast or iodine. After undergoing a discussion of procedures, consenting participants underwent a screening exam that included a urine pregnancy test and a pelvic examination with screening for chlamydia (Gen-Probe, San Diego, CA, USA) and evaluation of cervical patency (insertion of a uterine sound) to screen out participants who might not tolerate the repeated HSG examinations. Participants who reported more than minimal discomfort with sounding were excluded. Those meeting inclusion criteria with no exclusions were enrolled and instructed to use a reliable barrier method of contraception or to remain abstinent from sexual intercourse during the first cycle of participation. All participants received compensation.

Participants agreed to undergo at least three HSG procedures during the course of the study. The first two were scheduled during the first menstrual cycle after enrollment: one each during the follicular (cycle days 5–9) and luteal (cycle days 19–25) phase. After menses, participants received a COC (Portia® (30 mcg EE/ 150 mg LNG), TEVA Pharmaceuticals, North Wales, PA, USA) beginning on cycle days 1–5 and ingested one active pill daily (without a pill-free interval) until the third HSG was done 30 (± 2) days later.

**Fig. 1.**
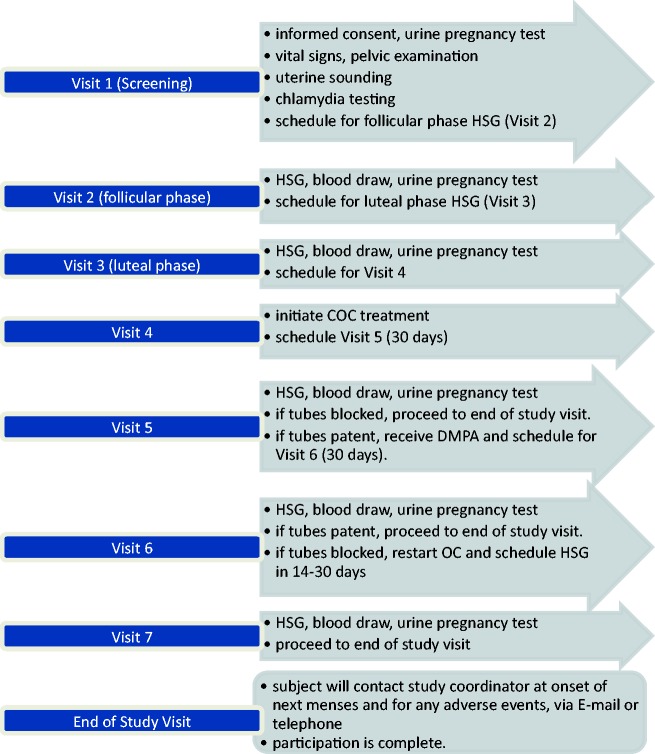
Visit content and study flow. COC, combined oral contraception; DMPA, depomedroxyprogesterone acetate; HSG, hysterosalpingogram.

Further participation depended on the tubal patency finding of the COC cycle HSG, ending if BTP was not observed. Participants with unilateral or bilateral patency received DMPA (depo-Provera®, Phamacia & Upjohn [Pfizer], New York, NY, USA) 150 mg IM and a fourth HSG was performed 30 (± 2) days later. Participation for the participant ended at this study if BTP was observed. If one or both tubes was occluded, participants reinitiated daily COC treatment until a fifth HSG 14–30 days later. After the final HSG, participants received contraception counseling and were asked to contact the study coordinator when they experience a return to normal menses (Fig. 1).

Prior to each HSG exam, participants submitted a urine pregnancy test, received single dose doxycycline 100 mg for antibiotic prophylaxis, and underwent blood sampling for hormone assays. One of the authors (AT), a board-certified woman’s radiologist, conducted all of the HSG examinations using standard techniques. With the participant in lithotomy, a vaginal speculum was introduced and the cervix cleansed with antiseptic solution. A flexible 5 F balloon catheter was introduced through the cervical os and inflated (either in the endocervical canal or in the uterine cavity) using 1 cc of air. Iohexol 300 mg/mL (Omnipaque®, GE Healthcare, Princeton, NJ, USA) contrast media was introduced using constant fluoroscopic guidance with spot images obtained during initial tubal filling, upon peritoneal spill, and to document important findings. When needed, prone and/or 10 min delayed images were obtained in an attempt to completely visualize the Fallopian tubes.

For the hormone levels, serum was separated from whole blood and frozen at −80℃. After completion of the study, specimens were shipped to the Endocrine Technology Services Core Laboratory (ETSL) at the Oregon National Primate Research Center (ONPRC, Beaverton, OR, USA; http://www.ohsu.edu/xd/research/centers-institutes/onprc/research-services/research-support/endocrine-technology.cfm) for analysis. The ETSL utilized the Roche Cobas e411 chemiluminescence-based automatic clinical platform (Roche Diagnostics, Indianapolis, IN, USA) to analyze samples for estrogen (E2, sensitivity 5 pg/mL) and progesterone (P, sensitivity 47 pg/mL). The intra- and inter-assay variation with the Roche Cobab e411 was consistently less than 10% for all assays. Quality control samples and validations were repeated prior to each assay run.

We hypothesized that the hormonal milieu influences functional tubal patency as assessed by HSG. Specifically, that BTP would be observed in all participants during the follicular phase, that some participants would show functional occlusion during the late luteal phase and following DMPA (low E2 and high progestin activity), and that patency would be restored in DMPA users treated with a COC therapy (high estrogen/progestin activity). We planned a pilot study to evaluate the feasibility of conducting repeated HSG examinations. Although no formal power calculation was performed, based on the 11% difference in patency observed between participants in the proliferative and secretory phase from the Lindahl study (2), we estimated that 10 participants would be the minimum number needed to observe at least one participant with a difference in functional tubal patency at one of the repeat exams.

## Results

A total of 11 participants signed informed consent. One participant failed screening as she was not able to tolerate placement of the uterine sound, and 10 enrolled in the study and completed the two natural cycle two HSG examinations. Table 1 lists the baseline characteristics of participants.

**Table 1.** Participant characteristics.

Participant characteristics (n = 10)	Mean ± SD
	Median (range)
Age (years)	34 ± 5.0
	35.5 (23–40)
BMI (kg/m^2^)	26.5 ± 5.3
	25.8 (20.0–34.1)
Ethnicity not Hispanic (n (%))	9 (90%)
Race (n (%))	
White	8 (80%)
Asian	1 (10%)
More than one race	1 (10%)
Nulligravid (n (%))	5 (50%)
One or more live birth (n (%))	4 (40%)

BMI, body mass index.

All 10 enrolled participants demonstrated BTP on at least one HSG examination during the natural cycle phase of the study (Table 2). One participant showed bilateral functional occlusion during the follicular phase examination, but BTP with the luteal phase, COC cycle, and DMPA exams.

**Table 2.** Results of HSG examinations and serum hormone levels during natural and contraception cycles.

	Participant A	Participant B	Participant C	Participant D	Participant E	Participant F	Participant G	Participant H	Participant I	Participant J
*HSG #1 follicular phase*										
Cycle day*	8	7	9	7	8	4	8	3	7	5
Right tube^†^	Blocked	Patent	Patent	Patent	Patent	Patent	Patent	Patent	Patent	Patent
Left tube^†^	Blocked	Patent	Patent	Patent	Patent	Patent	Patent	Patent	Patent	Patent
E_2_ pg/mL	36	66	12	29	57	81	72	34	251	72
P ng/mL	0.57	0.81	0.52	0.44	0.82	0.83	0.58	0.43	0.21	0.83
*HSG #2 luteal phase*										
Cycle day*	25	21	21	21	22	20	24	22	25	19
Right tube^†^	Patent	Patent	Patent	Patent	Patent	Patent	Patent	Patent	Patent	Patent
Left tube^†^	Patent	Patent	Patent	Patent	Patent	Patent	Patent	Patent	Patent	Patent
E_2_ pg/mL	52	29	117	86	199	55	191	61	135	100
P ng/Ml	1.71	11.77	7.64	0.26	18.28	2.41	20.36	5.85	14.18	11.07
*HSG #3 COC*										
Right tube^†^	Patent	Patent	Patent	DISCON	Patent	Patent	Blocked	Patent	Patent	Patent
Left tube^†^	Patent	Patent	Patent		Patent	Patent	Blocked	Patent	Blocked	Patent
E_2_ pg/mL	35	19	11		22	7	8	5	8	13
P ng/mL	0.94	0.67	0.76		0.71	0.87	0.51	0.5	0.32	0.77
*HSG #4 DMPA*										
Right tube^†^	Patent	DISCON	Patent		Patent	Patent	SPC	patent	Blocked	Patent
Left tube^†^	Patent		Patent		Patent	Patent		Patent	Patent	Patent
E_2_ pg/mL	52		15		81	41		28	15	21
P ng/mL	0.32		0.73		0.76	0.83		0.75	0.27	0.7
*HSG #5 COC cycle 2*										
Right tube^†^	SPC		SPC		SPC	SPC		SPC	Patent	SPC
Left tube^†^									Patent	
E_2_ pg/mL									8	
P ng/mL									0.2	

Follicular phase and luteal phase examinations performed according to participants’ self-report of menstrual cycle.

* Cycle day = number of days from first day of bleeding as reported by participant during natural cycle.

^†^Right tube and left tube assessment of patency blocked or patent.

COC, combined oral contraceptive; DISCON, participant discontinued study; DMPA, depomedroxyprogesterone acetate; E2, estradiol; P, progesterone; SPC, study protocol completed.

One participant with BTP on both natural phase studies discontinued participation, leaving nine who completed an exam while on the COC. Of these, seven showed BTP, one bilateral and one unilateral occlusion. Both of these participants demonstrated BTP during both exams in the natural cycle.

Participation ended for the participant with bilateral occlusion during the COC cycle, and one other declined DMPA injection, leaving seven participants who underwent an HSG exam 30 days after receiving DMPA. Of these, bilateral patency was observed in all participants, with the exception of the women with unilateral occlusion on the COC cycle; she continued to demonstrate unilateral occlusion, although this was the opposite side from the COC study. This participant restarted COCs and BTP was once again observed on the final HSG exam.

Table 2 shows the cycle day on which the natural phase HSG study was obtained according to the first day of last menses reported by the participant. We obtained serum samples for estradiol and progesterone to confirm cycle data. With the exception of participant 10 (E_2_ 251 pg/mL, P 0.21 ng/mL), who appeared to be mid-cycle, all of the follicular phase studies were consistent with menstrual dates. Similarly, with the exception of participant 5 (E_2_ 86 pg/mL, P 0.26 ng/mL), all of the luteal phase samples showed the anticipated elevation of progesterone. As expected, all of the participants that received the COC had estradiol in the menopausal range (<40 pg/mL) and P levels below 1 ng/mL suggesting ovarian suppression. Although P levels remained suppressed on DMPA, estradiol levels increased slightly in two participants (39–52 and 28–103 pg/mL). Of note, hormone levels were highly suppressed (E_2_ <10 pg/mL; P < 0.6 ng/mL) in the participants with unilateral or bilateral functional obstruction in both the COC and DMPA cycles.

## Discussion

The objective of this study was to determine whether functional tubal patency in women (as assessed by HSG) changes during the menstrual cycle or in response to hormonal contraception. Our long-term goal is to develop a low-cost, non-surgical approach to female permanent contraception. We have shown that a single treatment with 5% polidocanol foam results in a permanent collagen occlusion of the intramural Fallopian tube in non-human primates (7,8) and female baboons successfully treated do not become pregnant (9). However, not all females treated with polidocanol foam develop bilateral occlusion after a single treatment as is the case with quinacrine sterilization (3). A functional occlusion of the proximal tube that prevents movement of the active agent into the intramural Fallopian tube could explain some cases of treatment failure.

We had hypothesized that all of the studies in the follicular phase of the natural cycle would show BTP and expected that some of the luteal phase studies would show unilateral or bilateral blockage. Our results showed the opposite effect, with bilateral obstruction observed in one participant in the follicular phase that resolved in the luteal phase and was not seen with the COC or DMPA cycles. While based upon a small number of participants, our results support the recommendation of Lindahl to perform HSG in the luteal phase (2). However, most evaluations of tubal patency are performed during the workup for infertility and timing during the follicular phase offers the advantage of not disrupting a pre-implantation embryo.

Wanggren et al. performed repeated radionuclide hysterosalpingography in 10 healthy volunteers during the follicular, peri-ovulatory, and mid-luteal parts of the menstrual cycle in a design similar to our study (10). Although their findings were inconclusive, transport of radioactivity to the Fallopian tube was seen most frequently during the peri-ovulatory period, suggesting a hormonal effect influences tubal patency.

Prior to in vitro fertilization (IVF), considerable research interest revolved around the nature of proximal tubal obstruction observed on HSG (5,11). Sulak et al. (12) proposed that amorphous plugs (of undescribed origin) form a cast that obstructs the tube. Papaioannou (6) hypothesized that high smooth muscle tone and reduced ciliary activity during the follicular phase result in increased tubal secretions at the utero-tubal junction (UTJ) leading to stasis of the luminal contents and functional obstruction designed to delay the passage of the zygote in the ampulla for the first few days post conception. However, this hypothesis seems unlikely given the need for sperm to gain access to the upper reproductive tract for normal fertility.

Muscular contractility offers a more likely explanation for functional obstruction. The UTJ consists of three different smooth muscle layers; inner longitudinal, circular, and outer uterine spiral and shows a differential contractile response to prostaglandins during the menstrual cycle (13). Korenaga and Kadota found the contractile response to noradrenalin of circular muscle obtained from the isthmic Fallopian tube during the proliferative phase showed a high contractile response, while secretory phase samples were inhibitory (14). Lang described a series of 100 women evaluated in the follicular phase by HSG with blocked tubes (5). After treating a subset of these women for 6 days with aspirin, BTP was observed in 21 women. However, it is unclear if this effect was due to blocking prostaglandins or to the hormonal effect of examination later in the menstrual cycle.

The effects of exogenous hormonal therapy on functional occlusion are likely more complicated. When we treated baboons with DMPA 3–4 weeks prior to HSG, functional occlusion was common in females with low levels of estradiol (7). This suggests that estrogen action may be necessary prior to progesterone to induce progesterone receptor. Our results suggest that transcervical procedures for permanent contraception may be more successful when done at mid-cycle or in the early luteal phase.

A hormonally mediated blockade of the UTJ could represent an evolutionary adaptation to reduce the risk of endometriosis or ascending infection. The genital tract presents a vulnerability for female mammals, with a direct pathway to the peritoneal cavity. Maintaining a barrier would provide a selective advantage. However, the essential function of reproduction would require the UTJ to open prior to ovulation to allow sperm to enter the Fallopian tubes and to remain fully open through the mid-luteal phase to allow time for the fertilized embryo to enter the uterine cavity. When hormone levels drop prior to menstruation, the adaptive strategy would favor closure of the UTJ to prevent retrograde menstruation. The balance between reproductive and protective functions of the UTJ is illustrated by the expression of a variety of phenotypes. An imbalance leading to prolonged closure of the UTJ could result in infertility while the tendency toward prolonged patency could increase the risk of endometriosis.

Limitations of our pilot study include an insufficient sample size for statistical analysis. Although we attempted to obtain HSG examinations in both the follicular and luteal phase in all participants, hormone assays demonstrate that several participants were in the wrong phase. Although most participants showed evidence of suppression during the hormonal treatments, some may have been non-compliant with the COC. However, important strengths of our study include repeated exams in the same participants by the same radiologist and confirmation of BTP in all of the women that validates our findings of functional occlusion.

In conclusion, our small pilot study results support a role of hormonal action on the UTJ, with relaxation maximal following ovulation. Likewise, if tubal obstruction is observed during a follicular phase HSG performed for infertility, the study should be repeated during the luteal phase. Additional research investigating timing will be needed to confirm these observations.
